# Poly(N-isopropylacrylamide) based thin microgel films for use in cell culture applications

**DOI:** 10.1038/s41598-020-63228-9

**Published:** 2020-04-09

**Authors:** Ilaria Sanzari, Elena Buratti, Ruomeng Huang, Camelia G. Tusan, Franco Dinelli, Nicholas D. Evans, Themistoklis Prodromakis, Monica Bertoldo

**Affiliations:** 1 Faculty of Engineering and Physical Sciences, University of Southampton, Highfield Campus, Southampton, SO17 1BJ United Kingdom; 2 Istituto dei Sistemi Complessi del Consiglio Nazionale delle Ricerche (ISC-CNR), sede Sapienza, Pz.le Aldo Moro 5, 00185 Roma, Italy; 3 Istituto per i Processi Chimico Fisici del Consiglio Nazionale delle Ricerche (IPCF-CNR), sede di Pisa, via Moruzzi 1, 56124 Pisa, Italy; 4 Centre for Human Development, Stem Cells and Regeneration, Bioengineering Sciences, University of Southampton Faculty of Medicine, Tremona Road, Southampton, SO16 6YD United Kingdom; 5 Istituto Nazionale di Ottica del Consiglio Nazionale delle Ricerche (INO-CNR), via Moruzzi 1, 56124 Pisa, Italy; 6 Istituto per la Sintesi Organica e la Fotoreattivitá del Consiglio Nazionale delle Ricerche (ISOF-CNR), via P. Gobetti 101, 40129 Bologna, Italy

**Keywords:** Soft materials, Colloids, Biomaterials

## Abstract

Poly(N-isopropylacrylamide) (PNIPAm) is widely used to fabricate cell sheet surfaces for cell culturing, however copolymer and interpenetrated polymer networks based on PNIPAm have been rarely explored in the context of tissue engineering. Many complex and expensive techniques have been employed to produce PNIPAm-based films for cell culturing. Among them, spin coating has demonstrated to be a rapid fabrication process of thin layers with high reproducibility and uniformity. In this study, we introduce an innovative approach to produce anchored smart thin films both thermo- and electro-responsive, with the aim to integrate them in electronic devices and better control or mimic different environments for cells *in vitro*. Thin films were obtained by spin coating of colloidal solutions made by PNIPAm and PAAc nanogels. Anchoring the films to the substrates was obtained through heat treatment in the presence of dithiol molecules. From analyses carried out with AFM and XPS, the final samples exhibited a flat morphology and high stability to water washing. Viability tests with cells were finally carried out to demonstrate that this approach may represent a promising route to integrate those hydrogels films in electronic platforms for cell culture applications.

## Introduction

Smart hydrogels are three-dimensional polymer networks that can change their physicochemical properties in response to external stimuli, such as an electrical field and variations in temperature or pH. A great interest for these materials has emerged in the recent years, especially in the field of biomedical applications such as soft robotics^1^, drug delivery, cancer therapy^2^ and tissue engineering^3^ (TE). In TE, smart materials represent a promising option in order to develop *in vitro* models to mimic *in vivo* environments. For instance, it is known that diseased tissues present significant variations in their elastic behaviour^4^. Therefore electro-active polymers (EAPs), that can convert electrical signals into mechanical deformations and vice versa, would allow one to control the local stiffness of the substrates^5^.

Among the smart hydrogels employed in biomedical systems, poly(N-isopropylacrylamide) (PNIPAm) has deserved some special attention^6^. PNIPAm becomes hydrophobic and insoluble in water above a critical solution temperature (LCST), whereas it is soluble below this value^7,8^. The great interest for PNIPAm resides in the fact that its LCST value ranges between 32 to 35 °C, close to human body temperature^7,8^. This characteristic has been exploited, for instance, in cardiac TE to develop cell-sheet transplantation tissues from patient’s own autologous cells^6^. At 37 °C, PNIPAm surfaces are hydrophobic and cells can easily grow on them. When the temperature is reduced to 20 °C, PNIPAm becomes hydrophilic, promoting the detachment of cell-sheets^9–12^.

The addition of ionisable groups into a PNIPAm network in the form of a copolymer, semi-interpenetrating network (semi-IPN) or interpenetrating polymer network (IPN), has been already described as a possible strategy to improve the hydrogel elasticity and provide a multiresponvive ability to the systems^13,14^. In the presence of a relatively high concentration of ionisable groups along their backbone (anions or cations), the hydrogels are pH- as well as electro-responsive^15^. When a polyelectrolyte gel is placed in an electric field, free ions in the gel and surrounding solution begin to move towards the oppositely charged electrode. Consequently, the ionic distributions inside and outside the gel are not uniform. As the ionic concentration is one of the major repulsive factors affecting the gel volume, its variation inevitably results in a volume change and, hence, in a mechanical response of the sample.

In general, the response of polyelectrolyte gels depends on different parameters, such as their chemical nature, the salt species in solution, the sample position in relation to the electrodes and their size. Among other possible strategies to obtain multiresponsive systems, IPN shows better performances^13,16^. Consisting of two individual polymeric networks, that are physically entangled but not covalently bonded one to the other, the properties of IPN are additive. Thus elastic modulus can be modified while the mechanical properties of the two networks can be preserved^13,14^.

In spite of IPN’s potential, the application of stimuli responsive materials in TE is still limited due to the requirement for a complex synthesis, scarce processability, often poor mechanical properties, long response time and sometime inadequate biocompatibility^17,18^. However, many of the disadvantages above mentioned can be overcome due to recent techoological advances, i.e. employing micro- or nano-gels^19,20^. These hydrogels are a collection of nano- and micro-sized particles made of a crosslinked polymer. The hydrogels can be processed as polymer solutions due to their reduce size, to obtain thin films via solvent evaporation^21^, dip coating^9,22^, layer-by-layer assembly^23^ and spin coating^24–26^. Spin coating of colloidal microgel suspensions can represent a great advantage in order to obtain thin hydrogel films by offering more reproducibility and controllability of the process. However, thickness control and the possibility of patterning them with standard lithography have not been fully explored. In fact, hydrogels in the form of films are difficult to fix on substrates such as silicon wafer, glass or LiNbO_3_, as surface functionalisation is a very sensitive step. The advantage of having thin film smart hydrogels is connected with their response capabilities when subjected to different stimuli. Swelling/deswelling is a diffusion-based process, therefore in bulk hydrogels it may take a long time to occur after the stimuli are applied, resulting in a slow response. On the contrary, by decreasing feature size, such as in colloidal nanoparticles or thin films, it is possible to obtain a faster response^27^.

Recently, stable and smooth thin films of PNIPAm and PNIPAm/PAAc IPN have been prepared by spin coating of polymer solution, but not from a suspension of colloids. For example, Okano *et al*. have used PNIPAm thin films to detach cell monolayers without using proteolytic enzymes with the advantage of preserving adhesion proteins and thus grafting them to damages tissues such as cornea and heart^28^.

Herein, we describe a different strategy developed to prepare electro-active thin film hydrogels from nanogels suspended in colloidal solutions and anchor these films onto glass substrates. This stabilization strategy is based on chemical modification with vinyl moieties of previously sintetized microgel particles. In this way the hydrogels are chemically anchored to modified glasses via thermally activated thiol-ene reactions in the presence of dithiol molecules^29,30^. As predicted, this modification does not affect the other relevant properties. These films have been used as a platform for muscular cell cultures to investigate their potential integration in microfabricated electronic platforms to eventually control the stiffness or chemical surface of scaffolds for cell culturing applications.

## Experimental Section

### Reagents

N-isopropylacrylamide (NIPAm) was purchased from Sigma-Aldrich (purity 97%) and N, N’ methylene-bis-acrylamide (BIS) from East-man Kodak. They were recrystallized from hexane and methanol, respectively, dried under reduced pressure (0.01 mmHg) at room temperature, and stored at −20 °C until use. Acrylic acid (AAc) from Sigma-Aldrich was purified by distillation (40 mmHg, 60 °C) under nitrogen atmosphere in the presence of hydroquinone and stored at −20 °C until use.

From Sigma Aldrich we purchased: Sodium dodecyl sulfate (SDS), potassium peroxodisulfate, ammonium persulfate (APS), N,N,N′,N′-tetramethylethylenediamine (TEMED), 1-[3-(Dimethylamino)propyl]−3-ethylcarbodiimide methiodide (EDC methiodide), N-hydroxysuccinimide (NHS), 3-(Trimethoxysilyl)propyl methacrylate (TMSPMA) (purity 98%), 1,4-dithiothreitol (DTT), ethylenediaminetetraacetic acid (EDTA), NaHCO_3_, HCl 0.1 M and NaOH 0.1 M.

A dialysis tubing cellulose membrane with nominal molecular weight of 14 kDa from Sigma-Aldrich was washed as follows: 3 h in running distilled water, kept at 70 °C for 10 min in a aqueous solution containing NaHCO_3_ 3.0% wt and EDTA 0.4% wt, soaked in distilled water at 70 °C for 10 min, and finally washed in fresh distilled water at room temperature for 2 h. A float-A-Lyzer G2 dialysis device, SpectraPor, MWCO 100 kDa (Spectrum Laboratories, Inc.) was washed in 10% EtOH solution for 10 min and then in distilled water for 20 min.

Glass coverslips with a diameter of 13 mm, from Heinz Herenz Medizinalbedarf GmbH, were washed with acetone, isopropanol, deionized water and dried with nitrogen before using.

Ultrapure water was obtained using a Millipore DirectQ 3 UV purification system.

### Synthesis of the microgels

PNIPAm microgels were synthesized by a free-radical precipitation polymerisation of NIPAm in the presence of BIS as a crosslinker. In particular, 24.162 ± 0.001 g of NIPAm and 0.4480 ± 0.0001 g of BIS were dissolved in 1560 mL of ultrapure water containing 3.5190 ± 0.0001 g of SDS and transferred into a 2000 mL four-necked jacked reactor, equipped with a condenser and a mechanical stirrer.

The solution was purged by bubbling nitrogen for 1 h and then heated at (70 ± 1)°C. Polymerization was initiated after adding 1.0376 ± 0.0001 g of KPS, dissolved in 20 mL of deoxygenated water. The reaction was left to proceed while stirring at 70 °C for 5 h. The final product was purified by dialysis (MCWO 14 kDa) against distilled water daily refreshed for 2 weeks. The final weight concentration of the dispersion was 1.06%.

Poly(N-isopropylacrylamide-co-acrylic acid) P(NIPAm-co-AAc) microgels were prepared by a random co-polymerisation of NIPAm and AAc. In particular, 3.8107 g of NIPAm, 0.12 ml di AAc, 0.0711 g of BIS and 0.5815 g of SDS were solubilized in 250 mL of ultrapure water and transferred into a 500 mL four-necked jacked reactor, equipped with a condenser and a mechanical stirrer. The solution was deoxygenated by bubbling nitrogen for 1 h and heated at (70 ± 1)°C. A quantity of 0.1719 g of KPS, dissolved in 10 mL of deoxygenated water, was added to initiate the polymerization and the reaction was allowed to proceed at 70 °C for 22 h. The reaction product was purified by dialysis (MWCO 14 kDa) against distilled water with frequent water change for 2 weeks. The final concentration of the dispersion was 1.03% wt.

Interpenetrated polymer network (IPN) microgels were obtained using 140.09 g of the PNIPAm dispersion (1.06% wt), 5 mL of AAc and 1.1083 g of BIS. Acrylic acid (AAc) and BIS are added in the presence of a preformed PNIPAM particles swollen, in order to allow the growth of the PAAc network inside them, and to create the interpenetrated structure. The reagents were mixed into a 2000 mL four-necked jacketed reactor, diluted to 1260 ml with ultrapure water and deoxygenated by bubbling nitrogen for 1 h. A quantity of 0.56 mL of TEMED and of 0.4469 g of APS was then added to start the polymerization that was left to proceed for 3 h and 15 min at temperature lower than LCST (21 ± 1 °C). The final product was purified by dialysis (MWCO 14 kDa) against distilled water with frequent water change for 2 weeks, iced and lyophilized up to 1.00% wt concentration.

P(NIPAm-co-AAc)-ene microgels were prepared mixing 50.331 g of P(NIPAm-*co*-AAc) microgel (1.03% wt concentration), 0.0700 g of EDC and 0.0520 g of NHS, adjusting the pH to 4.5 with HCl 0.1 M. The mixture was stirred for 2 h, then 34 µl of allylamine was added and the pH adjusted to 10 by the addition of NaOH 0.1 M. The reaction was left to proceed for 48 h at room temperature. The modified microgels were purified by dialysis in two steps, first with a membrane with MWCO of 14 kDa, then with a membrane of 100 kDa against 0.1 M NaCl solution for 4 days and pure water for 4 days, with solvent changed twice a day. The purified dispersion was iced at −20 °C and lyophilized up to 3.00% wt concentration.

IPN-ene microgels were synthesized adding 0.1898 g of EDC and 0.1407 g to 30.452 g of IPN dispersion (1% wt concentration). The pH was adjusted to 4.5 with HCl 0.1 M, the mixture was stirred for 2 h; then 91 µl of allylamine was added and the pH was increased to 10 with NaOH 0.1 M. The modified microgels were purified by dialysis in two steps, first with a membrane with MWCO of 14 kDa, then with a membrane of 100 kDa against 0.1 M NaCl solution for 4 days and pure water for 4 days, with solvent changed twice a day. The final weight concentration of the dispersions was 0.35%.

### Glass treatments

Glass coverslips, employed as substrates, were treated in different ways. In one case, they were exposed to a freshly prepared “piranha” solution (a mixture of 96 vol% sulfuric acid and 30 vol% hydrogen peroxide heated at 120 °C for 5 min), rinsed with distilled water, and dried with nitrogen flow. The activated surfaces were then modified with a 3-(trimethoxysilyl)propyl methacrylate (TMSPMA) solution (100 mL deionised water, 10 μL acetic acid and 2% wt TMSPMA) overnight at 70 °C, and finally washed three times in ethanol and dried with nitrogen stream. Alternatively, the glass coverslips were immersed in a NaOH solution (10% w/w) at 80 °C in an ultrasonic bath for 5 min (Glass-NaOH). The substrates were then rinsed in hot water at 80 °C and dried under nitrogen flow.

### Film preparation

A volume of 1 ml of P(NIPAm-*co*-AAc)-ene (3% wt) and of 1 ml of IPN-ene (0.35% wt) dispersions was mixed with 63 µL and 19 µL of DTT (100 g/L in 1-butanol) in order to obtain P(NIPAm-*co*-AAc)-ene/DTT or IPN-ene/DTT mixtures. The films were made spin coating 60 µL of each mixture on TMSPMA-modified coverslips with a two steps procedure: first step, 30 s at 1000 rpm; second step, 60 s at 5000 rpm. The samples were then annealed in vacuum at 120 °C for 16 h, left to cool down to room temperature, washed with ultra-pure water, sonicated for 1 min, and dried with nitrogen flow. Reference films were prepared spin coating PNIPAm (3% wt) on piranha or TMSPMA-modified coverslips.

### Characterisation methods

Dynamic Light Scattering (DLS) analysis was carried out using a Zetasizer Nano ZEN1600 from Malvern Instruments (Worcestershire, UK), equipped with a He-Ne laser at a fixed angle of 135°. The measurements were carried out three times on freshly prepared diluted dispersions (∼0.02% wt) at 20 °C in a polystyrene cuvette. The hydrodynamic diameter, D_H_, of each microgel was obtained fitting the experimental data with the Kohlrausch-Williams-Watts expression, using the Stoke-Einstein equation^31^.

Infrared spectroscopy analysis was done using a Jasco FT/IR-6200 spectrometer, equipped with a Miracle ATR accessory, Pike Technologies, with ZnSe crystal. All the spectra were recorded on the lyophilized samples, accumulating 128 scans in the 4000–700 cm^−1^ spectral range. All the spectra were elaborated with Jasco Spectra Manager II, version 2.08.02.

^1^H NMR analysis was performed with a Bruker Avance DRX 400 spectrometer. Lyophilized samples were solubilized in deuterium oxide, (D_2_O) 99.9% atom of deuterium at 15 g/L, and analyzed at room temperature averaging over 256 scans.

For elemental analysis, the carbon and nitrogen contents were determined with a Flash EA1112, ThermoQuest/CE Elantech, Lakewood, NJelemental analyser on 1–5 mg of pulverized sample in sealed tin combustion boats at 900 °C. The evolved gases were analysed with gas chromatography on molecular sieves at 90 °C and a thermal conductivity detection system. Data acquisition and elaboration were done with the Thermo Quest CE Instrument Eager 300 Software version 1.01.

Contact angle analysis of the glass surfaces was carried out using a Drop Shape Analysis System (DSA 30 Kruss Co., Germany), equipped with a high-speed camera. A 5 µl drop of deionized water (R ~ 18 MΩ cm) was used. The shape of the drop was fitted with a polynomial function and the contact angle was calculated as average value of the right (θ_R_) and left (θ_L_) agles between the drop and the plane. All the values represent the average of measurements performed three times on three different samples.

AFM imaging was performed in tapping mode using either a Multimode Nanoscope V or a system made of a commercial head (SMENA, NT-MDT) with home-built electronics. Commercial Al-coated cantilevers (Budget Sensors) with resonance frequency about 300 kHz and force constant 10–130 N/m were used with Multimode Nanoscope V, whereas commercial HQ:NSC35 cantilevers (MikroMash) were employed with the customized AFM.

X-ray photoelectron spectroscopy measurements were performed using a Theta Probe System (Thermo Fisher Scientific INc., Loughborough, England) with Al–Kα source (photon energy = 1486.6 eV). set to illuminate a spot of 400 µm in size, whereas the vacuum chamber was pumped to 10^−9^ Torr. While measuring, an electron flood gun was used to prevent sample charging. Survey spectra were obtained with a pass energy of 160 eV and a step size of 0.1 eV. The elemental composition was obtained from the survey spectra after smart background subtraction. The spectra were also acquired in three different points for each sample in order to check the homogeneity of the films. Prior to spectrum acquisition an etching time of 20 s was carried out, in order to remove the traces of adventitious carbon. All the binding energies were calculated with respect to the peak of the C-C set at 285.0 eV^32^.

### Cell culturing assay

The glass coverslips were sterilised under UV light (254 nm) for 1 h in class I laminar flow hood. Afterwards, they were washed with warm (around 37 °C) ethanol 70% and subsequently washed 3 times in 0.5 mL of warm PBS. The samples were then placed in 24-well plates filled Dulbecco’s modified Eagle’s medium (DMEM) (Lonza), supplemented with 10% (v/v) fetal bovine serum (FBS) (Invitrogen), 1% (v/v) penicillin (P) and streptomycin (S) antibiotic (Lonza). The samples were maintained in a humidified incubator at 37 °C and 5% CO_2_. The glass coverslips treated with TMSPMA were used as control.

C2C12 cells (ECACC, Porton Down, UK) were cultured for 3 days in T125 flask (Corning, Sigma-Aldrich), up to 70–80% confluence. In order to split the cells, they were incubated with 2 μg/ml collagenase (Lonza) in serum-free DMEM (Lonza) media for 45 min in the humidified incubator at 37 °C and 5% CO_2_. Then the collagenase was removed, the cells were washed twice with PBS and incubated with trypsin (1×) (Lonza) for 5 min in the incubator. Then DMEM,10% FBS, and 1% P/S was added in order to inactivate the trypsin. The cell suspension was finally centrifuged at 200 *g* for 5 min. The cells were maintained in DMEM, 10% FBS, and 1% P/S were counted using a hemocytometer.

### Cell adhesion and activity

C2C12 cells were seeded at a density of 2000 cells/cm^2^ on 5 scaffolds in 24-well plates. Cell proliferation and adhesion on glass, glass/TMSPMA, glass/PNIPAm, glass/P(NIPAm*-co-*AAc)-ene and glass/IPN-ene were assessed monitoring the cells for 5, 24 and 72 h. A Live/Dead cell viability assay was used for a cell viability examination using Cell Tracker green (CTG) and ethidium Homodimer-1 (EH-1). Afterwards, the cells were seeded on scaffolds for 24 h, wells were washed and the media was removed. Reagents were subsequently added according to the manufacturer protocol (Life Technology). A volume of 300 μL of 10 μg/ml CTG and 200 μL of 5 μg/ml EH-1 in serum-free DMEM media were added to each well, and placed in the incubator for 1.5 hrs. The plate was then removed from the incubator, the media was replaced with DMEM media, and incubated for 45 min. Then the cells were washed, fixed in PFA and imaged. Images of the scaffolds were taken using an inverted microscope (Nikon Eclipse, Nikon UK, Surrey, UK), equipped with a fluorescence filter. Cell adhesion and spreading were evaluated capturing images in bright-field at two different magnifications (4× and 20×). The cell density was evaluated using ImageJ (NIH, Baltimore) and processing images at a magnification 4×. The background of each image was subtracted and the cells were counted automatically using the Analyse particles plugin. The cell spreading area was measured using the same software for images at a magnification 20×, and the outline was drowned manually around each cell. The fluorescent staining was imaged at a magnification 10x using two filters, red (Ex 530–560 nm, Em 570–640 nm) and green (Ex 450–490 nm, Em 500–550 nm). Three images per sample were taken in different areas, and three samples per category were analysed.

## Results and Discussion

### Synthesis and characterization of vinyl-functionalized microgels

To prepare stimuli-responsive surfaces immobilized on a rigid substrate, we selected the following strategy: synthesis of microgels based on NIPAm and AAc; partial substitution of the carboxylic acid groups with vinyl groups; functionalization of glass surfaces with vinyl groups; film deposition spin coating dispersions with the presence of dithiol molecules; promotion of grafting among particles and between particles and glass surface via thermally activated thiol-ene reactions^30^.

**Figure 1:**
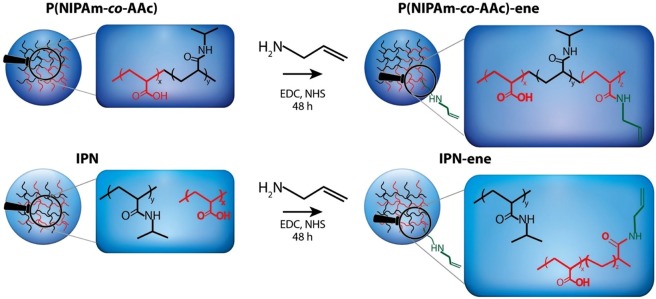
Schematic representation of the functionalization of P(NIPAm-*co*-AAc) and IPN microgels with –ene reactive groups. The P(NIPAM-co-AA) and IPN nanogels are is ene-functionalized by allylamine at room temperature in the presence of EDC/NHS couple. Then, allylamine is added to the for the formation of the amide bonds onto the carboxylic acid. The reaction is allowed to proceed for 48 h.

Two different microgels made of NIPAm, AAc and BIS, namely P(NIPAm-*co*-AAc) and IPN, were prepared by copolymerization and sequential polymerization, respectively^13,19,33–35^. The amount of acid moieties in the purified products, as determined from ^1^H-NMR and elemental analyses, was 9.2 and 38.9 mol%, respectively (see Supporting Information)^35^. Part of these groups was modified in the following reaction step by amidation with allylamine (Fig. 1) in presence of EDC and NHS acting as dehydration and addition promoter, respectively^30,36^. It is known that the insertion of repeating units different from NIPAm may affect the response of the whole system, in particular in the case of random copolymers^33,34^. Furthermore, the convertion of a large number of acid groups would result in a loss of the pH rensponsiveness. Thus, our goal was inserting not more than a few percent per mole of vynil groups, in order to preserve both the temperature and pH stimuli-responsive behaviours. The amidation reaction was carried out with a reduced amine concentration in the feed (amine/acid = 0.9), even though the reaction is known not to bequantitative^37^.

**Figure 2:**
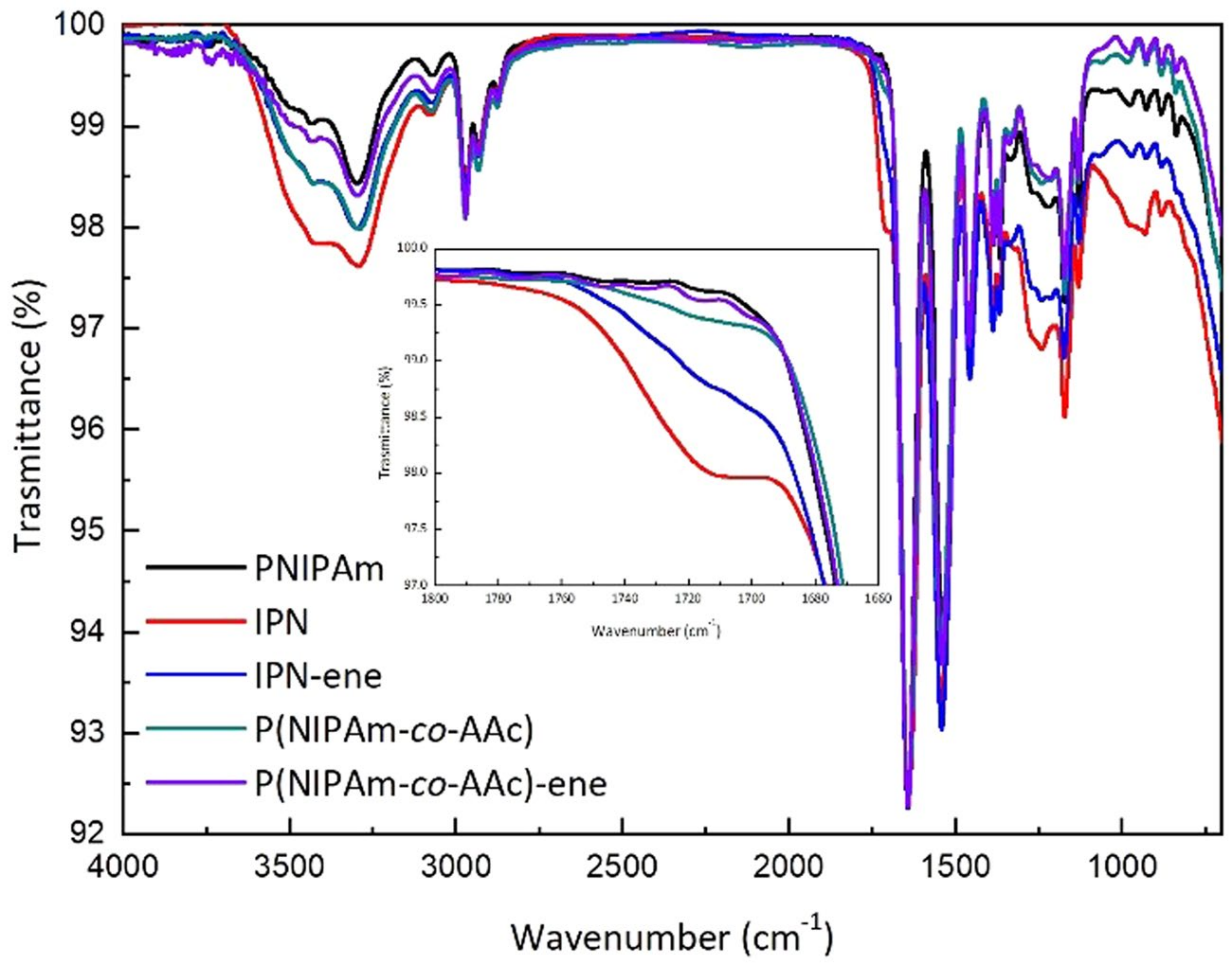
ATR FT-IR spectra of the NIPAm based microgels before and after the amidation reaction with allylamine. Inset: a zoom of the peak at 1725 cm^−1^, to show the carboxylic acid moiety of AAc. The spectra are normalized with respect to the adsorption band at 1645 cm^−1^, due to the PNIPAm network.

The final products were analysed with FT-IR spectroscopy (Fig. 2). An intensity decrease of the band at 1725 cm^−1^, due to the C=O of the carboxylic acid moieties (inset in Fig. 2), was observed for both the modified microgels. This evidence shows that the desired reaction had occurred in both cases. Unfortunatly, the presence of the absorption band of the NIPAm amide groups prevents the view of possible new peaks due to the formation of the covalent bonds with the vinyl amine moieties. In any case, no new carbonyl signal, attributable to an intermediate reaction or side products (such as for instance NHS esters), was detected. This indicates the effectiveness of the performed purification steps.

**Figure 3:**
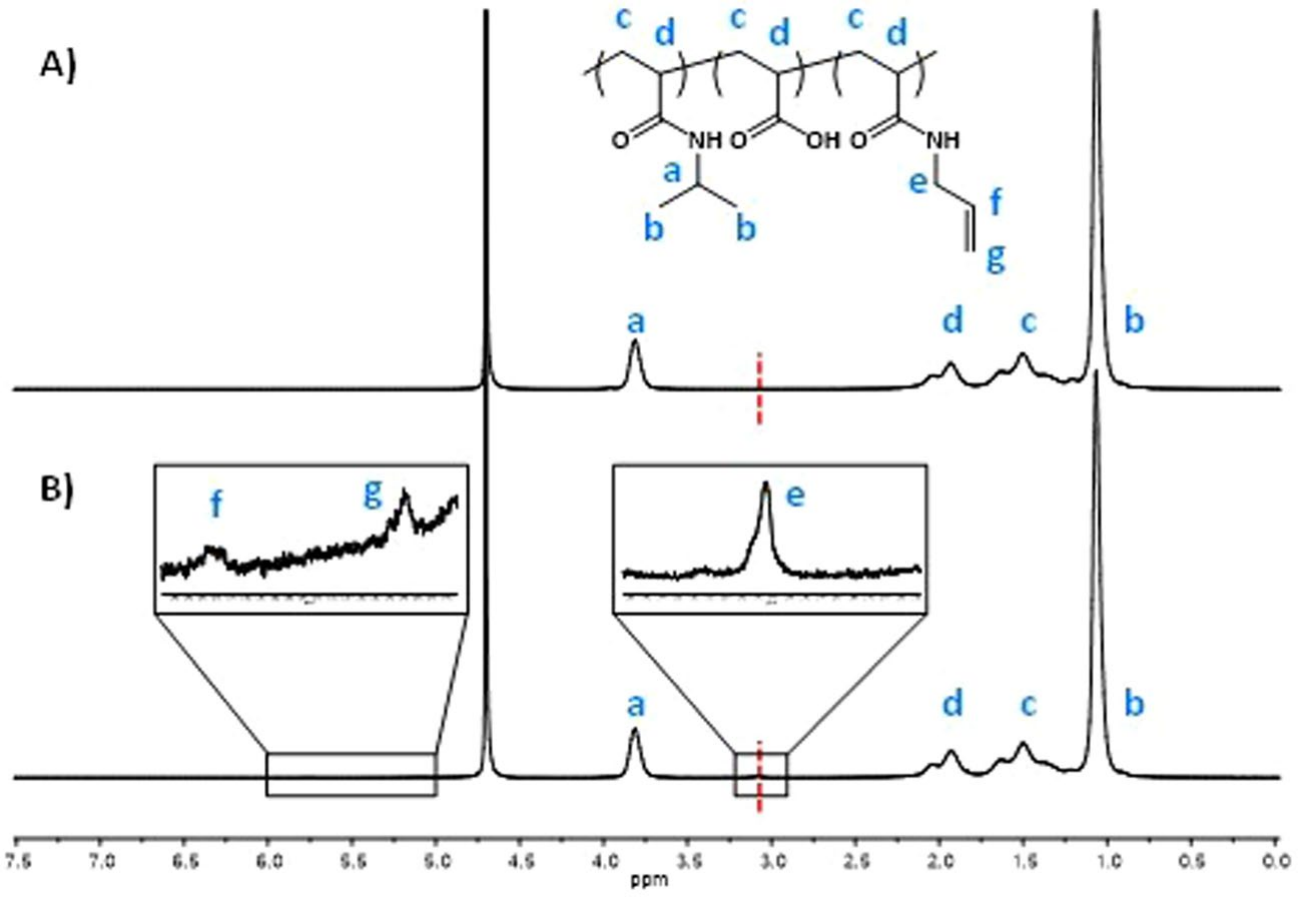
1H-NMR spectra of P(NIPAm-co-AAc) (**A**) and P(NIPAm-co-AAc)-ENE (**B**) microgels in D_2_O. The chemical structure represents the P(NIPAm-co-AAc)-ene microgel. Insets in (**B**) right, a zoom of the peak at 3.07 ppm due to the vinyl moiety; left, a zoom of the peak at 5.5 ppm. The spectra are normalized with respect to the peak at 3.82 ppm, due to the isopropyl group of the PNIPAm network.

Additional evidence that the desired reaction had occurred, was obtained by ^1^H-NMR spectroscopy. A comparison between the spectra of the pristine and modified microgels shows the signals of the polymer main chain (*c* and *d*) and of the NIPAm isopropyl groups (*a* and *b*) in all samples (Fig. 3 and S1). In addition, in the modified microgels three new small peaks can be observed at 3.07, 5.10 and 5.77 ppm. In the presenting order, they can be attributed to protons of the methylene group of vinyl moieties (*e*), protons of substituted vinyl carbon (*f*) and two protons on the therminal vinylic carbon (*g*).

A quantification of the double bonds actually inserted, *ENE* (% mol), was obtained from Eq. (1), where A_e,_ A_a_ and A_bcd_ are the areas of the signals at 3.07, 3.8 and in between 0.75 and 0.25 ppm, respectively. The amount of the repeating units per mole in the polymeric network bearing a vinyl functionality is 1 and 2% for the random copolymer and IPN, respectively (Table 1).

1 $$ENE( \% \,mol)=\frac{3{A}_{e}}{2({A}_{bcd}-6{A}_{a})}\cdot 100$$

**Table 1:** Composition of the ene-functionalized microgels and their precursors, as determined from combining ^1^H-NMR and elemental analyses^35^.

	NIPAM (% mol)	AAc (% mol)	BIS (% mol)	Allylamine (% mol)	Diameter (nm)	
					20 °C	40 °C
PNIPAm	99.98	0	0.02	0	70 ± 5	43 ± 1
IPN	54.8	38.9	6.3	0	406 ± 36	367 ± 27
IPN-ene	56.8	34.9	6.3	2.0	478 ± 20	436 ± 25
P(NIPAm-*co*-AAc)	89.6	9.2	1.2	0	236 ± 30	102 ± 23
P(NIPAm-*co*-AAc)-ene	89.5	8.3	1.2	1.0	424 ± 30	541 ± 400

In the last column their respective size, as determined from dynamic light scattering measurements.

In both cases, many carboxylic acid groups were left unmodified. This amount (*AAc*) was calculated from Eq. (2) assuming that all groups in the pristine microgels that were not converted into vinyl amide groups were still in the acid form: roughly four time larger for IPN-ene than for P(NIPAm*-co-*AAc)-ene. *NIPAm* and *BIS* represent the values obtained for the pristine microgels, as previously described (Table S1)^35^.

2 $$AAc( \% \,mol)=100-NIPAm-BIS-ENE$$

The size of the particles in a hydrated state was determined with dynamic light scattering (Fig. S2). IPN at 20 °C presents a larger size than PNIPAm, from which it was synthetized (Table 1). This can be expected due to the insertion of a quite large amount of the second network based on PAAc (see combined ^1^H-NMR and elemental analysis data in Table 1 and Supporting Information, Fig. S1 and Table S1).

After the modification of IPN with allylamine, the particle size becomes only moderately larger. Actually, even if the amount of inserted functionality is very low and no significant weight change should be expected, the particle size depends on other parameters, such as the hydratation degree and the interparticle interaction. Therefore, these data indicate a modest effect of the ‘ene’ modification on these last mentioned parameters, and the preservation of a colloidal stability of the microgel dispersion.

The pristine P(NIPAm-*co*-AAc) and the derived ene-modified microgels have an intermediate particle size with respect to PNIPAm and IPN. Additionally, both samples are characterized by a very broad particle size distribution (β ∼ 0.53). The parameter β, which is 1 for monodisperse particles and almost zero for infinite broadened distribution, is instead ∼ 0.88 for IPN and ∼ 0.93 for PNIPAm. Unlikely IPN-ene, the functionalization changes the particle size thus indicating a large effect of the modification in the interparticle interaction. This effect is particularly evident at 40 °C where particles tend to aggregate (see correlation plot shape). Any possible responsiveness of the system is masked by the interparticle interaction.

In the case of both IPN and IPN-ene a modeste change of size was observed by heating the dispersions from 20 °C to 40 °C. In this case, the apparent lack of responsiveness is due to the PAAc cloud surrounding the particles. Even though the PNIPAM core collapses, the particle shell made of PNIPAM is scarcely affected, leaving the whole diameter almost unchanged. This effect has been previously reported for PNIPAM/PAAc IPN with a large amount of PAAc, like the one here investigated^31^.

### Activation of the glass surface

**Figure 4:**
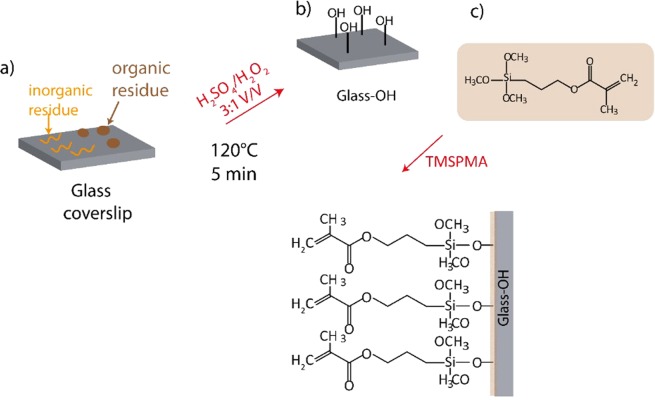
Scheme of the reaction carried out to functionalize the glass coverslips with vynil groups. Organic residues can be removed by washing the substrates in piranha solution (**a,b**), and then silanized with TMSPMA (**c**).

In order to provide the substrate with double bonds for the immobilization of the microgels, 3-(trimethoxysilyl)propyl methacrylate (TMSPMA) was selected as silanization reagent. The modification was performed treating first the coverslips with a piranha solution to expose hydroxyl groups on the glass surface. Figure 4 shows the silanisation steps and the chemical bonds exposed on the surface of glass after the treatment.

**Figure 5:**
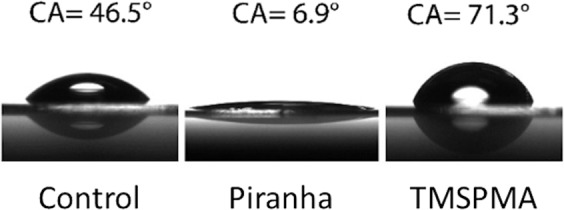
Contact angle (CA) measurements for a glass coverslip not treated (control), after piranha treatment and after TMSPMA silanization.

Contact angle (CA) measurements were carried out to asses that each chemical modification had successfully taken place. The control value, measured on a clean coverslip before any treatment, was found to be 47 ± 1°. After the treatment with piranha solution, CA decreased to 6 ± 2° due to the hydrolysis of the Si-O-Si bonds with the formation of Si–OH groups. These functionalities provide the glass surface with both hydrophilicity and reactivity towards silanizing agents. After the TMSPMA treatment, the CA value increased to 70 ± 4° (Fig. 5). TMSPMA, even though it cannot be considered a very hydrophobic moiety, has a rather hydrophobic character if compared with the hydroxilic groups. The treatment with NaOH provided a value of 36° ± 2°, only moderately lower than pristine glass.

### Preparation of the microgel films

**Figure 6:**
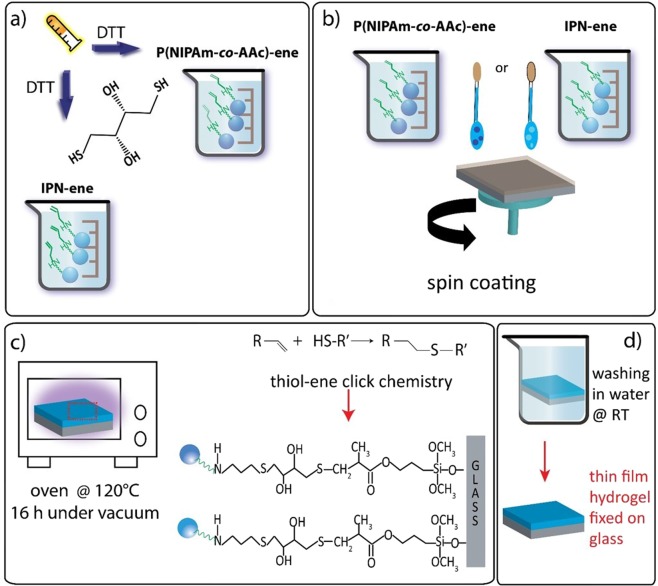
Preparation scheme of anchored hydrogel films: ene-functionalized colloidal suspensions with the addition of DTT (**a**); spin coating on glass coverslips functionalized with TMSPMA (**b**); thiol-ene click reactions activated in oven at 120 °C (**c**); washing in water at room temperature and final drying (**d**).

The final step is an adaption of a previously reported recipe, employed for the immobilization of a linear P(NIPAm-*co*-AAc) copolymer on a glass surface^30^. Dispersions of P(NIPAm-*co*-AAc)-ene/DTT and IPN-ene/DTT, 3 and 0.35% wt respectively, were spin coated on glass-TMSPMA according to the optimized conditions reported in a previous work (Fig. 6). A PNIPAm dispersion 3% was also spin coated as control. The addition of DTT has the goal to promote grafting of the particles on the modified glass substrate and crosslinking of the particles among themselves. The presence of two thiol (S-H) groups on each DTT molecule allows the reaction with the vinyl functionalities present on both the substrate and the particles (Fig. 6)^29,31^.

A stabilisation occurs if the two thiol groups of a DTT molecule reacts with two vinyl groups belonging to different particles or a particle and the glass surface. The concentration chosen for PNIPAm and P(NIPAm-*co*-AAc)-ene was 3% wt^30^. For IPN-ene, the concentration was limited to 0.35% due to the high viscosity of the dispersion. Actually, IPN-ene exhibits a higher viscosity than pristine IPN, most likely due to the interaction between the amine and carboxylic acid functionalities belonging to distinct particles. The two complementary functionalities may form strong hydrogen bonds between them.

**Figure 7:**
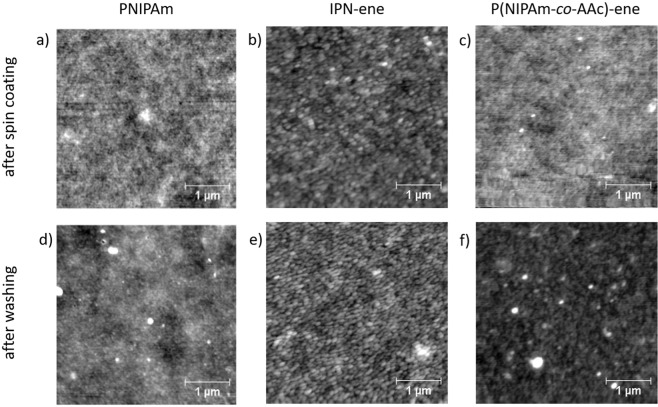
Top row: AFM images of pristine PNIPAm, IPN-ene and P(NIPAm-co-AAc)-ene films (**a–c**, respectively). Bottom row: AFM images after thermal treatment and washing of PNIPAm, IPN-ene and P(NIPAm-co-AAc)-ene films (**d–f**, respectively).

In spite of that, continuous films with morphologies similar to PNIPAm were obtained with both ene-modified microgels (Fig. 7). The topography is a nearly flat with no detectable signature of the individual particles. This indicates that the ene-modification does not significantly affect the film formation.

**Figure 8:**
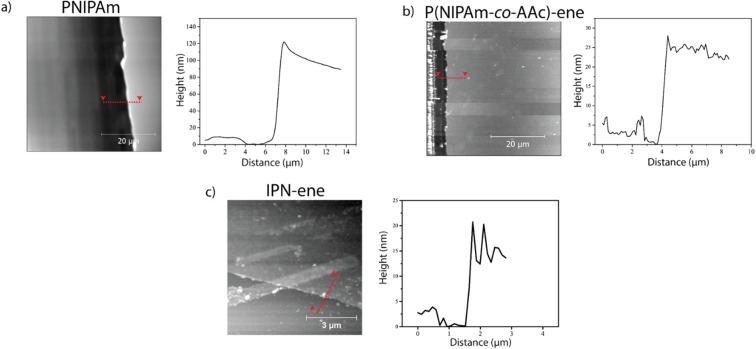
AFM images and height profiles along the edges of the cuts for PNIPAm (**a**), P(NIPAm-co-AAc)-ene (**b**) and IPN-ene (c) films.

The calibration of the thickness was made creating a scratch in the middle of each sample and measuring step profiles of the cut (Fig. 8). The height values were: 14 ± 2.0 nm for IPN-ene; 30 ± 6 nm for P(NIPAm*-co-*AAc)-ene; 150 ± 12 nm for PNIPAm. IPN-ene films were prepared from a dispersion 10 times more diluted than PNIPAM and P(NIPAm*-co-*AAc)-ene; accordingly, its thickness resulted the lowest. Nerapusri *et al*. reported a thickness value comparable to the particle size for PNIPAm and P(NIPAm-*co*-AAc), when spin coat ing a dispersion 0.5% wt^9^. Therefore, the presence of a layer, only one particle thick, can be assumed for IPN-ene film. In the case of P(NIPAm*-co-*AAc)-ene, the thickess of the film is larger than the one of IPN-ene in spite to the smaller size of the particles. However, the larger size can be explained if one takes into account the largest concentration of the solution used for spin coating. Finally, the highest film thickness was observed for PNIPAM. This sample has the lowest particle diameter and the solution concentration for spin coating was comparable to the one of P(NIPAm*-co-*AAc)-ene. Theferore, a stacking of many one-particle layers can be invoke to explain the observed results. The ability of PNIPAm to create many one-particle layer films, unlike the microgels that contains AAc units, can be explained by the absence of repulsive forces, present in the case of IPN-ene and P(NIPAm*-co-*AAc)-ene.

The lower thickness of films with respect to the particle diameters (Fig. S2 and Fig. 8) can be explained by the high degree of deformability of the particles. This hypothesis is supported by the morphology of an IPN-ene sample made with drop casting (as reported in Fig. S3), which shows the ability of the particles to flatten. The same behaviour was previously reported for other weakly crosslinked microgels of 1:1 copolymers of N-Vinylcaprolactam and NIPAm^30^.

The grafting and crosslinking processes were promoted by annealing the films at 120 °C for 16 h. Heat is needed to activate and to speed up the reactions by increasing the mobility of the reagents^30^. An AFM analysis showed no significan variation in morphology for both IPN-ene and P(NIPAm*-co-*AAc)-ene after the heat treatment (Fig. 7). The glass transition temperature of PNIPAm in the dry state is around 135–142 °C^38–40^, and increases when copolymerized with AAc^41^. It is known that below the glass transition temperature only atom vibrations and some reorganization of small side groups on the local scale may occur. Therefore, for an annealing temperature below the glass transition, only a minor morphological evolution can be expected.

**Figure 9:**
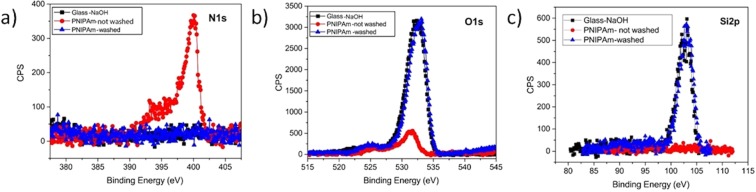
High resolution XPS spectra: N1s (**a**), O1s (**b**) and Si2p (**c**) peaks obtained for glass treated with NaOH, pristine and washed PNIPAm films.

To assess the immobilization of the films, the samples were washed in water at room temperature and then analysed with AFM and XPS. PNIPAm before washing showed XPS peaks due to carbon (C1s) at 275–290 eV, nitrogen (N1s) at 390–403 eV and oxygen (O1s) at 520–536 eV, in agreement with the elemental composition of the organic layer that is made of carbon, nitrogen, hydrogen and oxygen (Fig. 9). Notice that the peak of silicon (Si2p) at 102.4 eV was not detected after coating; this indicates a good coverage of the whole substrate. On the other hand, the silicon peak was detected for the pristine glass-NaOH substrate before and after washing. In agreement with some previously reported results, PNIPAm after washing was not detectable anymore; the topography is similar to pristine glass (Fig. 7). Thus PNIPAm on an activated glass substrate is unstable in water at room temperature^30^. Furthermore, after washing the signal of carbon and nitrogen were not detectable, thus confirming a complete removal of the organic film. The O1s peak position at 532.4 eV for the washed film and at 531.5 eV for the non-washed one is consistent with the absence of organic matter (only O atom in O-Si bonds) in the first case and of the organic layer containing the C=O groups of PNIPAm in the latter one^42^.

An AFM analysis of IPN-ene and P(NIPAm*-co-*AAc)-ene after washing showed that the organic layers were still present with roughness of almost 550 pm, even though the film morphologies did not exactly match those ones observed before washing (Fig. 7). In particular, an increased roughness was observed, most likely due to a relaxation occurring during the washing.. Films after spin coating had a flat morphology with roughness of almost 700 pm.. This morphology after spin coating may be due to the ability of the microgels to squeeze and stretch during the spinning process. Furthermore, the microgels used in this work are very soft because of the very low degree of crosslinking, intentionally used to promote interparticle overlapping.

**Figure 10:**
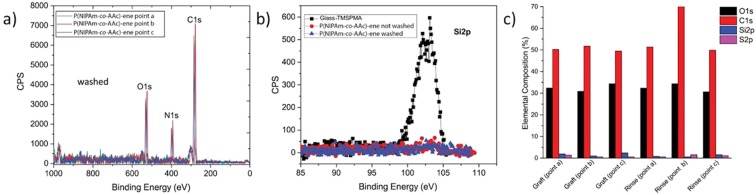
XPS spectra of P(NIPAm-co-AAc)-ene (**a**). In (**b**) Si2p spectra of glass treated with TMSPMA and of a P(NIPAm-co-AAc)-ene film before and after washing. In (**c**) a bar graph with the elemental composition of P(NIPAm-co-AAc) films grafted and rinsed. The measurements were taken in different areas of the same sample.

A homogeneus coverage of the substrate with P(NIPAm-*co*-AAc)-ene was confirmed from XPS showing a C1s intensity comparable in different areas after spin coating (Fig. 10). After annealing and washing, the C1s and N1s core level peaks were still observed even if their spatial homogeneity was worse, in agreement with the AFM observations. Furthermore, the Si2p signal was not detected both after spin coating and after washing, thus confirming a full coverage of the substrate with the P(NIPAm-*co*-AAc)-ene. Low intensity peaks at 164.0 eV, corresponding to organic sulphur (C-SH or C-S-C)^43^, were also detected in both washed and not-washed P(NIPAm-*co*-AAc)-ene films. The detection of sulfur is a fingerprint of DTT, since microgels do not contain any sulfur. The presence of DTT is clearly expected before washing, whereas its permanence afterwards suggests that the addition reaction of all vinyl moieties has occurred.

**Figure 11:**
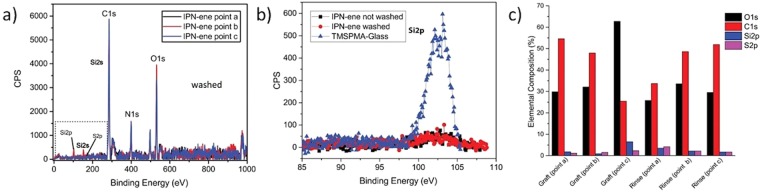
XPS spectra of IPN-ene (**a**). In (**b**) Si2p spectra of glass treated with TMSPMA and of an IPN-ene film before and after washing. In (**c**) a bar graph with the elemental composition of IPN-ene films grafted and rinsed. The measurements were taken in different areas of the same sample.

For IPN-ene, continuous flat films were observed both before and after washing with surface roughness of 600 pm before an after washing (Fig. 7). This was confirmed from XPS, even though data indicate a less homogeneus coverage after washing with respect to P(NIPAm*-co-*AAc)-ene (Fig. 11). The reason for such a behaviour may be attributed to a lower thickness of the IPN-ene film compared to the P(NIPAm*-co-*AAc)-ene one. This results in a higher probability of film discontinuity in case a few particles are washed off.

Finally, the AFM and XPS data suggest that, even when the microgels are washed, some organic material still persists. In order to further explore this observation, IPN-ene films were prepared on a Parylene C masked substrate (Fig. S4). After washing, the Parylene C mask was peeled-off and a replica was still observable on the substrate, thus confirming the persistence of a thin film after washing. The edge of the film allows one to evaluate its thickness, which was around 10 nm before and after washing, in agreement with the results obtained from the analysis of the step profiles. This experiment provides clear evidence of the film persistency and shows the potentiality of the strategy we propose for microfabrication.

### Cell culturing

**Figure 12:**
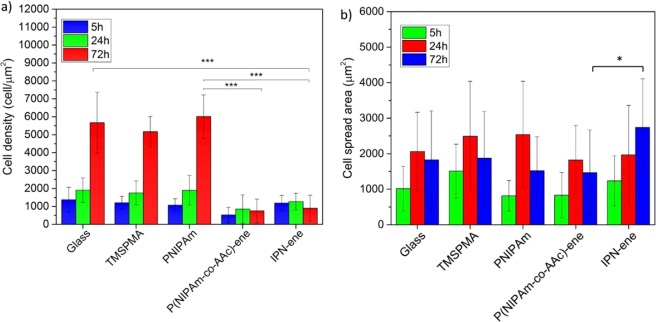
Cell proliferation and spreading after three different incubation intervals: 5, 24, 72 h. The number of cells is plotted in (**a**), the cell spread area in (**b**). Data reported are mean values ± standard error, *p < 0.5, ***p < 0.001.

C2C12 were cultured on P(NIPAm-co-AAc)-ene, IPN-ene compared to glass, TMSPMA and PNIPAm controls. Cells proliferated on glass, glass-TMSPMA and PNIPAm, as shown by an increase in cell number with respect to time (Fig. 12a). However, proliferation was significantly reduced on either P(NIPAm-co-AAc)-ene or IPN-ene. At 72 hours, the number of cells attached to glass, glass-TMSPMA and PNIPAm was similar, but but was significantly lower for P(NIPAm-co-AAc)-ene and IPN-ene. The cells were generally well spread on controls and PNIPAm. On IPN-ene cells were significantly more spread than on P(NIPAm-co-AAc)-ene as shown in Fig. S5 and 1Fig. 12.

**Figure 13:**
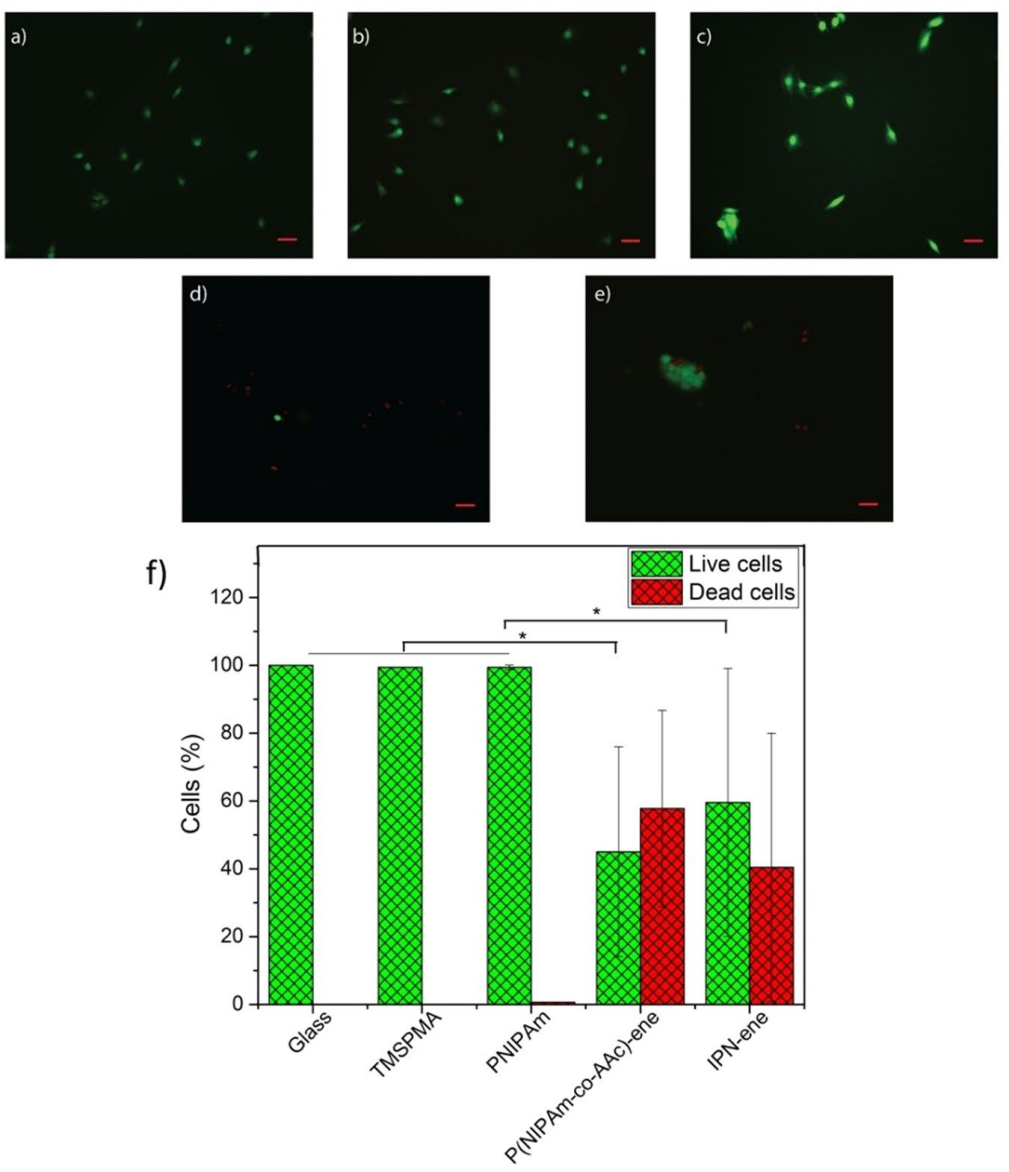
Live/dead assay images for cell viability of cells grown on pristine glass, glass/TMSPMA, PNIPAm, P(NIPAm-co-AAc)-ene and IPN-ene (named IPN). In (**a–e**) we show fluorescent live/dead assay images representing cell viability. In (**f**) a statistical analysis of a number of cells dead and the number of cells alive is reported. All data represent the mean values ± standard error, *p < 0.5. Red is for dead cells and green for living cells. The initial seeding density was 20000 cells/cm^2^ and the incubation time 24 h. Red scale bar: 50 µm.

As compared to controls, where almost no cell death was evident at 24 hours, cell cultures on P(NIPAm-*co*-AAc)-ene or IPN-ene contained significant numbers of dead cells (53.9% on P(NIPAm-*co*-AAc)-ene and 40.4% on IPN-ene respectively as shown in Fig. 13). The results for PNIPAm are in good agreement with the literature, showing cell proliferation above the LCST where surfaces are hydrophobic^44–46^. On the contrary, the presence of AAc in P(NIPAm-*co*-AAc)-ene and IPN-ene provides the material with a pH sensitive character, negative charge and strong hydrophilicity at neutral (7) and basic pH (>7) due to the dissociation of AAc in -COO^−^ (above pK_a_ = 4.5)^14^. As result, since cells are negatively charged due to the glycolipids and glycoproteins located on their membrane, electrostatic cell–substrate interactions is not facilitated and cells do not adhere on the substrates because of charge repulsion^47^.

We have also observed that cells could live in some areas but not in others, even though IPN-ene contains the largest amount of AAc. To demonstrate that, we have drop casted IPN-ene on a glass coverslips and then we have seeded the cells. Since the hydrogel is not uniform there are some thinner and thicker areas. From Fig. S6 in the Supporting Information, it is evident that cells prefer to live in thinner regions rather than thick areas. For this reason, it can be hypothized that IPN-ene is so thin that cells may sense the underlying TMSPMA-glass, so that they cannot proliferate as they do on pristine TMSPMA-glass.

In summary, both P(NIPAm-*co*-AAc)-ene and IPN-ene resulted cytotoxic. The cytotoxicity of these films can be attributed to the presence of AAc. It was already demonstrated that P(NIPAm-*co*-AAc) can induce a different proliferation depending on the amount of AAc present in the gel. When this value approaches 4%, the cell growth is gradually inhibited because the films becomes more and more hydrophilic and the cell/substrate interaction is reduced^48^. However, cells seems to slightly favour IPN-ene, in spite to the higher amount of the carboxilic groups: about 40% for IPN and 10% for the copolymer.Therefore, other factors must be taken into consideration; for example, the different chemical structure possibly results in a different exposure of the AAc groups, or the different particle density may result in a different elastic modulus or a different thickness of the film. It is known that the elastic modulus of the substrate has a profound effect on cell spreading^49^, as well as the film thickness^10,50^. On the other hand, in the case of the IPN structure the location of the AAc moieties in the second interpenetrated network may promote a masking effect with a consequentially lower cytotoxicity. In this respect, further studies need to be undertaken in order to understand the effects on the surface properties due to the interpenetrated structure, such as the weight ratio between the two networks, or the size of the particles and the crosslinking degree.

## Conclusions

Microgels based on NIPAm and AAc were synthesized and modified with vinyl moieties. Uniform and continuous films were obtained by spin coating suspensions of the microgels on glass substrates, previously modified with vinyl groups. Thin films were successfully anchored to the substrates via post-deposition heat treatment in the presence of dithiol molecules.

Preliminary cell growth tests were performed using muscular cells. A negative result on cell viability was observed, attributed to the presence of acrylic acid. However, the toxicity was surprisingly larger for random P(NIPAm-*co-*AAc) compared to IPN, the latter containing four times more AAc. Provided the need of an optimization of the AAc content in order to avoid any toxicity effect, IPN may represent a promising candidate for substrate patterning in cell culture applications.

## Supplementary Information

Supporting Information.

## Data Availability

All data supporting this study are available from the University of Southampton repository at: 10.5258/SOTON/D0952
