# Supplementary material for: Poly(N-isopropylacrylamide) based thin microgel films for use in cell culture applications
Source: Sci Rep. 2020 Apr 9;10:6126. doi: 10.1038/s41598-020-63228-9 (PMC7145875; doi:10.1038/s41598-020-63228-9)
Supplement: Supplementary file 1 — Supporting Information. [file 41598_2020_63228_MOESM1_ESM.docx]

Supporting Information

**Poly(N-isopropylacrylamide) based thin microgel films for use in cell culture applications**

Ilaria Sanzari^1*^, Elena Buratti^2,3^, Ruomeng Huang^1^, Camelia G. Tusan^4^, Franco Dinelli^5^, Nicholas D. Evans^4^, Themistoklis Prodromakis^1^ and Monica Bertoldo^3,6^

^1^ Faculty of Engineering and Physical Sciences, University of Southampton, Highfield Campus, Southampton, SO17 1BJ, United Kingdom

^2^ Istituto dei Sistemi Complessi del Consiglio Nazionale delle Ricerche (ISC-CNR), sede Sapienza, Pz.le Aldo Moro 5, 00185 Roma, Italy

^3^ Istituto per i Processi Chimico Fisici del Consiglio Nazionale delle Ricerche (IPCF-CNR), sede di Pisa, via Moruzzi 1, 56124 Pisa, Italy

^4^ University of Southampton, Centre for Human Development, Stem Cells and Regeneration, IDS/Somers Building, Faculty of Medicine, Southampton, SO16 6YD, UK

^5^ Istituto Nazionale di Ottica del Consiglio Nazionale delle Ricerche (INO-CNR), via Moruzzi 1, 56124 Pisa, Italy

^6^ Istituto per la Sintesi Organica e la Fotoreattivitá del Consiglio Nazionale delle Ricerche (ISOF-CNR), via P. Gobetti 101, 40129 Bologna, Italy

E-mail: ilaria.sanzari@gamail.com

**^1^H-NRM analysis on IPN and IPN-ene**

**Figure S1.** ^1^H-NMR spectrum of A) IPN and B) IPN-ene microgels in D_2_O. The chemical structure represents IPN-ene microgel. The insets in B) are a zoom of the peaks at 3.07 ppm due to the vinyl moiety. Spectra are normalized with respect the peak at 3.82 ppm due to the isopropyl group of PNIPAm network.

**Elemental analysis**

**Table S1.** Content of nitrogen, carbon, hydrogen and oxygen for PNIPAm, IPN, P(NIPAm-co-AAc) obtained from elemental analysis.

|  | **N**  **(% mol)** | **C**  **(% mol)** | **H**  **(% mol)** | **O**  **(% mol)** |
| --- | --- | --- | --- | --- |
| **PNIPAm** | 11.2 | 57.7 | 10.3 | 20.8 |
| **IPN** | 7.3 | 43.7 | 7.7 | 41.3 |
| **P(NIPAm-*co*-AAc)** | 10.9 | 58.3 | 9.7 | 21.1 |

**DLS analysis on microgels**


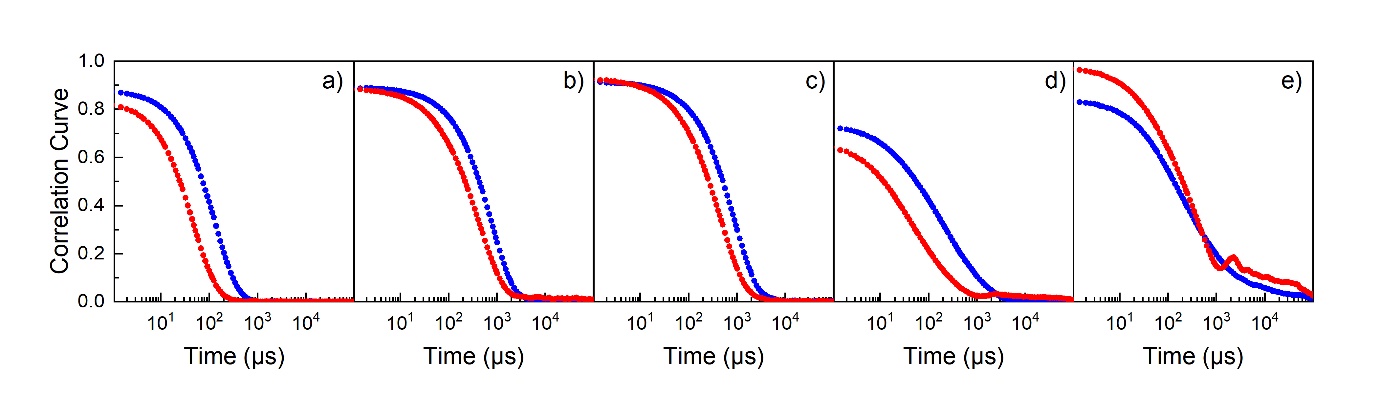


**Figure S2.** Correlation curves of a) PNIPAm, b) IPN, c) IPN-ene, d) P(NIPAm-*co*-AAc) and e) P(NIPAm-*co*-AAc)-ene. The analysis was performed at a concentration of 0.02 g/L. The blue lines are referred to analysis performed at 20 °C and the red ones to analysis performed at 40 °C.

### **Drop casting of diluted IPN-ene NPs**


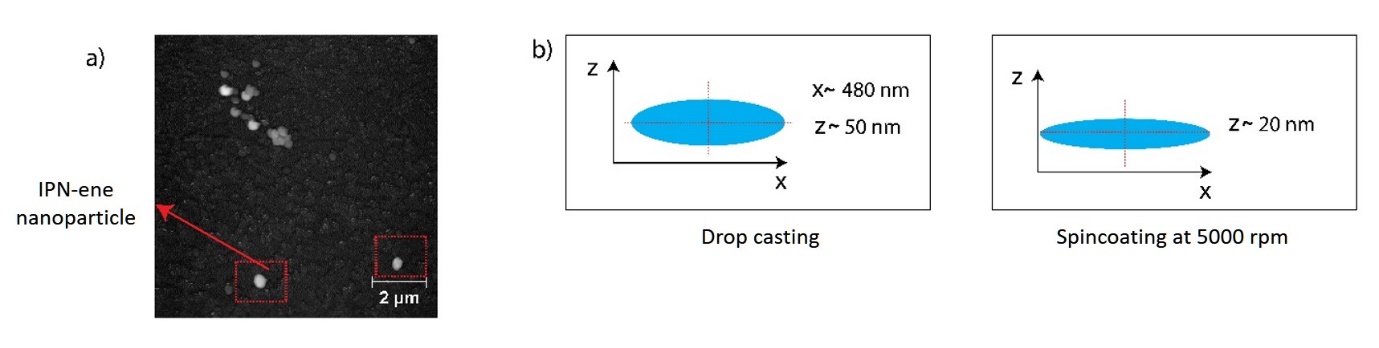


**Figure S3**. In (a) is shown an AFM image of IPN-ene nanoparticle drop casted on a glass coverslip (a). In (b) the difference between the shape of nanoparticles dip casted (left panel) and spin coated (right panel) is depicted. It is hypothesized that the nanoparticles are more stretched when spin coated with a short axis with a lower dimension than the short axis on the nanoparticles dip casted.

**Spin coating of IPN-ene using Parylene-C mask**


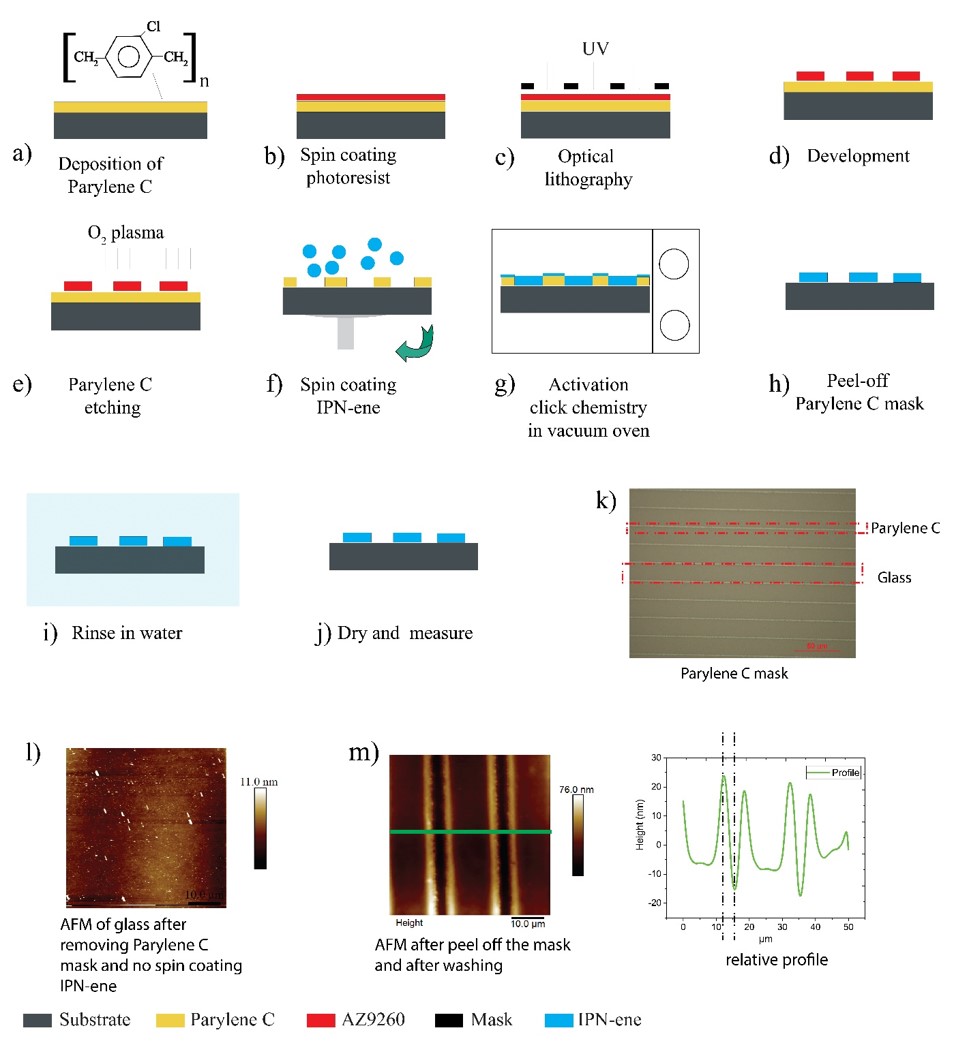


**Figure S4.** 5 µm thick Parylene C was coated on a borosilicate wafer (a). AZ9260 positive resist was spin coated on Parylene C with thickness of almost 6.5 µm (b). Successively, optical lithography was performed with exposure time of 20 sec (c). After development in AZ400k:water (1:3) for 3 min and 30 sec (d), the wafer was exposed to oxygen plasma in ICP (1000W ICP power) for almost 9 min (etching rate of Parylene 600 nm/min) (e). The wafer was then cut in 1cmx1cm square and silanized with TMSPMA. 60 µl of IPN-ene colloidal solution were then spin coated on the Parylene C mask and put in a vacuum oven for 16h at 116 °C to activate the click chemistry (g). Parylene C mask was then peeled-off (h) and the samples with IPN-ene were washed several times in water (i). Samples were successively dried, and AFM was performed to see if there was a film with the pattern of Parylene C mask (j). The optical micrograph of the mask is shown in (k). The AFM topography of the glass substrate after the peel-off of the mask is shown in (l). After peeling off the mask and washing the sample, a pattern that follow the mask was found out. In (m) the AFM image of the patterned IPN-ene film with the relative profile is reported.

**Bright-field microscopy images of C2C12 cells**


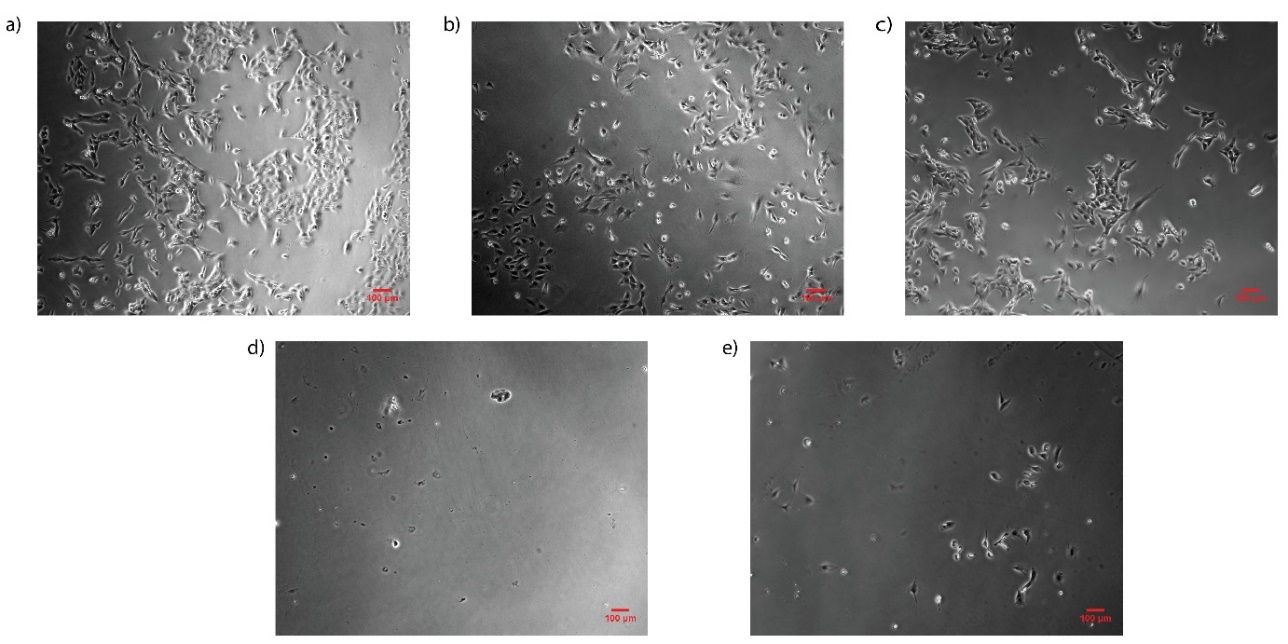


**Figure S5**. Bright-field microscopy images of C2C12 cells cultured for 72 h on glass treated with piranha (a), glass treated with TMSPMA (b), PNIPAm (c), P(NIPAm-co-AAc)-ene (d), IPN-ene (e). Red scale bar: 100 µm


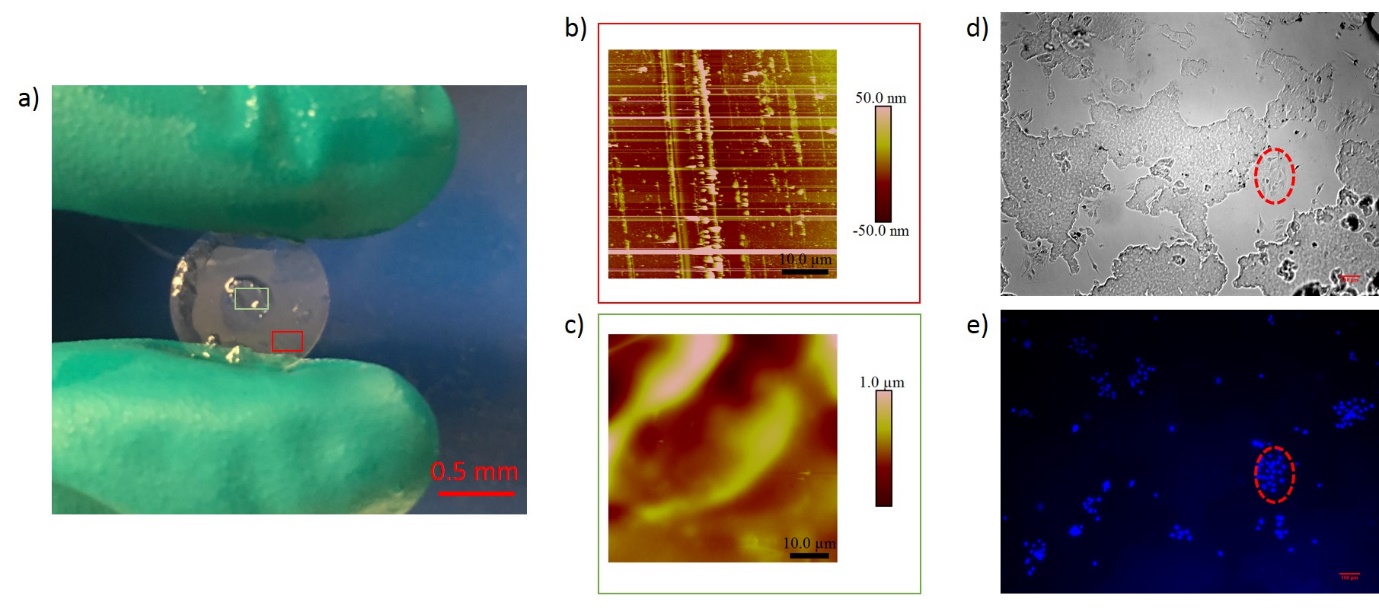


**Figure S6**. Images of solvent casted hydrogel on glass coverslips with AFM topographies of thin and thick areas. In (a) a picture of the IPN film in swollen state. In (b) and (c) the corresponding AFM topographies of step created in thin (red panel) and thick areas (green panel) of the sample. In (d) bright field and the corresponding fluorescent image (DAPI) (e) of C2C12 seeded for 24 h at density of 2800 cells /cm^2^. The red circles show cells that leaves in thinner areas. (Red scale bar: 100 µm).
